# Progesterone and Estrogen Receptors in Neurofibromas of Patients with NF1

**DOI:** 10.4137/cpath.s1002

**Published:** 2008-09-15

**Authors:** Mauro Geller, Spyros G.E. Mezitis, Fabio Pereira Nunes, Marcia G. Ribeiro, Alexandra Prufer de Q.C. Araújo, Marcello D. Bronstein, Rodrigo Siqueira-Batista, Andréia Patrícia Gomes, Lisa Oliveira, Karin Soares Gonçalves Cunha

**Affiliations:** 1 Teresópolis Medical School (UNIFESO)—Teresópolis, Rio de Janeiro, Brazil; 2 Federal University of Rio de Janeiro—Rio de Janeiro, Rio de Janeiro, Brazil; 3 New York-Presbyterian Hospital/ Weill-Cornell Medical Center and Lenox Hill Hospital—New York, NY, U.S.A; 4 Massachusetts General Hospital, Harvard University, Charlestown, MA, U.S.A; 5 Hospital das Clínicas (USP)—São Paulo, São Paulo, Brazil; 6 Universidade do Grande Rio—Rio de Janeiro, Rio de Janeiro, Brazil

**Keywords:** neurofibroma, plexiform neurofibroma, progesterone receptor, estrogen receptor

## Abstract

Neurofibromatosis type 1 (NF1) or von Recklinghausen disease is a genetic disorder affecting the growth of cells in nervous system. One of the most remarkable characteristics of this disease is the development of benign tumors of the nervous system (neurofibromas).

The purpose of this study was to test tissue samples taken from neurofibromas and plexiform neurofibromas of NF1 patients for the presence of estrogen and progesterone receptors. We used previously collected samples from patients registered in the database of the *Centro Nacional de Neurofibromatose* (CNNF-Brazil). Samples from twenty-five patients in the database presenting plexiform neurofibromas (N1 group) and 25 samples from the same database from patients presenting neurofibromas (N2 group) were tested.

We observed positive staining for progesterone receptors in 13 of the neurofibroma samples and 19 of the plexiform neurofibroma samples. Among the neurofibroma samples, we observed one sample with positive estrogen receptor staining, but none of the plexiform neurofibroma samples showed positive staining. We suggest further studies to investigate in greater depth possible hormonal influences on the development and growth of neurofibromas and plexiform neurofibromas in NF1.

## Introduction

Neurofibromatosis type 1 (NF1) is an autosomal dominant disorder primarily affecting the cell growth of neural tissue. The disorder is caused by a mutation affecting the gene located on chromosome 17q11.2, occurs in an estimated 1:3000 births, with considerable variation in clinical presentation and disease severity among affected individuals (Gutmann et al. 1997; DeClue et al. 2000; Cunha et al. 2003; Geller and Bonalumi Filho, 2004; Geller et al. 2006). The clinical signs and symptoms of NF1 may be present at birth or may develop at any age (Theos and Korf, 2006). Common features of NF1 include Lisch nodules, café-au-lait spots, freckling in the inguinal and axilary regions and neurofibromas (Ferner, 2007). Multiple neurofibromas tend to develop in NF1 patients, but solitary neurofibromas may affect individuals that do not have NF1 (Geller and Bonalumi Filho, 2004; Theos and Korf, 2006).

One of the most visible signs of NF1 is the dermal neurofibroma, a benign, heterogenic tumor of the nervous system composed of Schwann cells, perineural cells, fibroblasts, mast cells, and axons in an extracellular matrix (De Clue et al. 2000; Zhu et al. 2002; Ferner, 2007). Neurofibromas may be focal growths or can extend along the length of a nerve and may arise on any part of the body, cutaneously or subcutaneously (Theos and Korf, 2006). While cosmetic alterations arising from dermal neurofibromas are among the chief complaint among NF patients, cutaneous neurofibromas may also cause itching and stinging, and subcutaneous neurofibromas may be painful and cause neurological deficit as a result of nerve compression (Ferner, 2007).

Plexiform neurofibromas are classified as benign peripheral nerve sheath tumors. They are not metastatic, but are highly vascularized and involve multiple nerve fascicles, often making surgical excision difficult (Korf, 1999). Plexiform neurofibromas can occur congenitally, although deeper tumors may not be recognized until later in life (Geller and Bonalumi Filho, 2004; Theos and Korf 2006). Plexiform neurofibromas can be very large and irregularly shaped, and may cause neurological deficit due to compression of adjacent structures, as well as soft tissue and bony hypertrophy (Korf, 1999; Theos and Korf, 2006).

The actions of estrogenic hormones mediated through the estrogen receptor (ER) play important roles in regulation of growth, differentiation, and function of many reproductive organs. Estrogens increase proliferation and alter cell properties, in part, via induction of growth factor receptor and growth factor. In addition ER has an interrelationships with the progesterone receptor (PR) system in modulation of responses (Katznellenbongen, 1996). Progesterone promotes regulation of some tissue remodulation and, like other sex steroid hormone receptors, is expressed in vascular cells (Weigel, 1996).

Some authors suggest an increase in the size and number of neurofibromas as well as an increased potential for malignant transformation of plexiform neurofibromas during periods of hormonal change, specifically puberty and pregnancy (Puls and Chandler, 1991; Posma et al. 2003). These observations indicate a possible influence of steroid hormones on the development of neurofibromas (Dugoff and Sujansky, 1996; Geller et al. 2006). The steroid hormone receptors belong to a large group of nuclear ligand-activated transcription factors which play a key role in transcriptional activation as well as several aspects of biological function, including reproduction, metabolism, and regulation of development. Hormone binding triggers a series of events that result in the activation or repression of target genes (Weigel, 1996).

This study was part of the neurofibromatosis research line subdivided in immunogenetic therapeutic investigation supported by reference centers in Brazil, together with Harvard Medical School^2^. The purpose of this study was to observe the presence of progesterone and estrogen receptors in the neurofibromas and plexiform neurofibromas of male and female patients with NF1.

## Materials and Methods

Following Ethical Committee approval (approval no.19/04), we performed a descriptive case study on 50 NF1 patients registered in the database of the Centro Nacional de Neurofibromatose (CNNF- Brazil), a national reference center for NF. Twenty-five patients were selected for each group among patients in the database presenting plexiform neurofibromas (N1 group) and neurofibromas (N2 group). The inclusion criteria for this study required patients to be diagnosed with NF1 in accordance with the diagnostic criteria established by revised NIH criteria, be registered in the CNNF database, and have stored tumor material from a previous biopsy. Patients presenting a clinical history of malignant tumors were excluded from the study. A careful review of each patient’s medical history was performed using information stored in the CNNF database.

Serial 5-mμ sections were cut from paraffin wax blocks and collected on silane coated slides. After dewaxing, the presence of ER and PR were demonstrated by routine immunohistochemistry using the LSAB method (LSAB® + KIT, code K0690, Dako Corporation, California, U.S.A). Antigen retrieval was performed using microwave ovens and citrate buffer (pH 6.0) in a pressure cooker. Endogenous peroxidase activity was eliminated by incubation for 10, 15 and 20 minutes in 6% H_2_O_2_ in distilled water at room temperature. Non-specific protein binding was blocked by incubation with a 1:100 dilution of normal goat serum in antibody diluent with background reducing component (code S3022; Dako Corporation, California, U.S.A), for 30 minutes at 37 °C. Sections were incubated overnight at 4 °C with a 1:30 dilution of the primary monoclonal antibody against ER (clone 1D5; code N1575; Dako Corporation, California, U.S.A.) and with a 1:200 dilution of the primary monoclonal antibody against PR (clone 16; code RTU-PGR-312; Novocastra^™^, Newcastle Upon Tyne, UK). Visualization was performed by incubation for five minutes in diaminobenzidine. Between each step, sections were washed three times for 10 minutes in Tris buffered saline. All incubations were carried out in humidified chambers to prevent evaporation. Sections were counterstained in Mayer’s haematoxylin and they were coverslipped with Entellan® (code 107961; Merck©, Darmstadt, Germany). Negative controls were performed by omission of the primary monoclonal antibody. A breast carcinoma was used as positive control.

A semiquantitative counting was carried out in the region with the highest number of stained cells. Samples were classified as having “no staining” if <5 positive cells were found per 10 high-power fields, and “positive” if there were >5 positive cells/10 high-power fields. Fisher’s exact test was used to compare results within and between patient groups.

## Results

The N1 group (plexiform neurofibromas) was comprised of 13 male patients and 12 female patients, while the N2 group (cutaneous, subcutaneous, pendular neurofibromas) included 10 male patients and 15 female patients. Mean patient age in the N1 group was 25 (+10.65), while the mean age in the N2 Group was 18 (+13.37).

**Figure 1.**
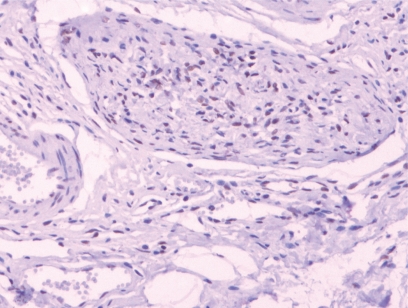
Plexiform neurofibroma with nuclear staining for progesterone receptor. 20x.

Table 1 summarizes the results of the analysis. We observed positive progesterone receptor staining in the tumor samples of 13 patients of the N1 group, of which 7 samples were from male patients and 6 were from female patients, with no statistically significant between-gender difference in PR staining (p = 1.000) (Fig. 1). In the N2 group, we observed positive PR receptor staining in 19 samples, of which 7 were from male patients and 12 from female patients. No statistically significant difference in the number of PR-positive samples was observed between the male and female patients of this group (p = 0.653). A comparison of the number of PR-positive tumor samples from both groups did not present statistically significant difference (p = 0.139).

**Figure 2.**
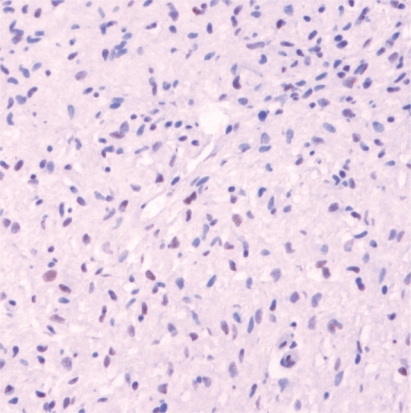
Neurofibroma with immunopositive cells for estrogen receptor. 20x.

Positive ER staining was observed in only one sample, from the N2 group, belonging to a female patient. This sample did not present positive staining for progesterone receptor (Fig. 2).

## Discussion

We observed positive PR staining from both neurofibroma and plexiform neurofibroma samples, with no significant difference based on gender within each group or between the two groups. Of the 50 tumor samples analyzed, only one presented positive ER staining. These findings correlate with those presented by McLaughlin and Jacks (2003) who found that out of 59 neurofibromas and plexiform neurofibromas analyzed for ER and PR, 75% were positive for PR while only 5% stained positively for ER. Our findings show 64% (32/50) of the tumor samples analyzed with positive PR staining and 2% (1/50) positive for ER.

Although their role remains debated, steroid hormone receptors have been observed in numerous other human tumor types, including primary spinal cord tumors, small-cell lung cancer, and solitary pelvic neural tumors, and melanomas (Grill et al. 1981; Concolino et al. 1984; Chetkowski et al. 1985; Kaiser et al. 1996). In the case of neurofibromas and plexiform neurofibromas, the observation of an increase in number, size, and malignancy potential—specifically in plexiform neurofibromas—during puberty and pregnancy suggest a hormonal influence in the development, growth, and distribution of these tumors. In a survey of 59 NF1 patients using hormonal contraception, Lammert et al. (2005) found only 5 patients reporting neurofibroma growth during contraception^20^. However, as pointed out by the authors, the study was based on *a posteriori* questionnaires. Additional studies to determine the presence of neurofibroma growth in terms of size, mass and tumor depth are warranted to confirm Lammert’s suggestion that hormonal contraceptives may not stimulate neurofibroma growth.

Fishbein et al. (2007) compared steroid hormone receptor expression and ligand-mediated cell growth in normal human Schwann cells with neurofibroma-derived Schwann cells. They reported differential expression of ER, PR, and androgen receptor in primary neurofibromas and neurofibroma-derived Schwann cells, as compared to unaffected Schwann cells. However, expression did not correlate with different neurofibroma types, and differences were observed between culture samples and primary tissue samples. Changes in terms of proliferation and apoptosis were found to be heterogenous among groups and ligands, and were considered to be consistent with increased cell accumulation. The authors suggest a direct influence of the steroid hormones, through their receptors, in the initiation and progression of neurofibromas.

The extremely variable clinical expressivity of NF1 makes it a challenging disorder for the clinician and the researcher. Cutaneous neurofibromas are among the chief complaint of NF1 patients for cosmetic reasons, while plexiform neurofibromas may present periods of rapid growth, causing disfiguration and compression of surrounding structures, with severe cases presenting as life threatening. To date, treatment remains surgical, with complete resection of plexiform neurofibromas difficult in many cases due to infiltration around nerve roots (Korf, 1999). The suggestion by Fishbein et al. that future therapy may be individualized for each tumor based on its steroid hormone characteristics, highlights the importance of these and other studies in order to gain a greater understanding of the factors contributing to neurofibroma growth in a step towards the development of alternative treatment for these difficult tumors.

The results of this study confirm the presence of progesterone receptors in both neurofibromas and plexiform neurofibromas in patients with NF1. The presence of these receptors may account, at least in part, for the periods of rapid growth and proliferation of neurofibromas and plexiform neurofibromas observed in many NF1 patients, particularly during puberty and pregnancy.

## Figures and Tables

**Table 1.** Gender distribution of immunopositivity for Progesterone Receptor (PR) and Estrogen Receptor (ER) by patient group.

	**N1 Group**		**N2 Group**	

	**PR**	**ER**	**PR**	**ER**
Male	7/13	0/13	7/10	0/10
Female	6/12	0/12	12/15	1/15
Total	13/25	0/25	19/25	1/25

## References

[b1-cpath-1-2008-093] Chetkowski R, Sakamoto H, MacLusky N (1985). Solitary pelvic neural tumors with high steroid receptor content. Gynecol. Oncol..

[b2-cpath-1-2008-093] Concolino G, Liccardo G, Conti C (1984). Hormones and tumors in central nervous system (CNS): steroid receptors in primary spinal cord tumors. Neurol. Res..

[b3-cpath-1-2008-093] Cunha KSG, Barboza EP, da Fonseca EC (2003). Identification of growth hormone receptor in localised neurofibromas of patients with neurofibromatosis type 1. J Clin Pathol.

[b4-cpath-1-2008-093] DeClue JE, Heffelfinger S, Benvenuto G (2000). Epidermal growth factor receptor expression in neurofibromatosis type 1-related tumors and NF1 animal models. J. Clin. Invest..

[b5-cpath-1-2008-093] Dugoff L, Sujansky E (1996). Neurofibromatosis type 1 and pregnancy. Am. J. Med. Genet..

[b6-cpath-1-2008-093] Ferner RE (2007). Neurofibromatosis 1. Eur J Hum Genet.

[b7-cpath-1-2008-093] Fishbein L, Zhang X, Fisher LB (2007). In vitro studies of steroid hormones in neurofibromatosis 1 tumors and schwann cells. Mol. Carcinogenesis.

[b8-cpath-1-2008-093] Geller M, Bonalumi Filho A (2004). Neurofibromatose: Clínica, Genética e Terapêutica.

[b9-cpath-1-2008-093] Geller M, Ribeiro MG, Araújo APQC (2006). Serum IgE levels in neurofibromatosis 1. Int J Immunogenet.

[b10-cpath-1-2008-093] Grill HJ, Benes P, Manz B (1981). Steroid hormone receptors in human melanoma. Arch Dermat Res.

[b11-cpath-1-2008-093] Gutmann D, Aylsworth A, Carey J (1997). The diagnostic evaluation and multidisciplinary management of neurofibromatosis 1 and neurofibromatosis 2. Jama.

[b12-cpath-1-2008-093] Kaiser U, Hofmann J, Schilli M (1996). Steroid-hormone receptors in cell lines and tumor biopsies of human lung cancer. Int. J. Cancer.

[b13-cpath-1-2008-093] Katznellenbongen BS (1996). Estrogen Receptors: Bioactivities and Interactions with Cell Signaling Pathways. Biol Reproduction.

[b14-cpath-1-2008-093] Korf B (1999). Plexiform Neurofibromas. Am. J. Med. Genet. (Semin. Medical Genetics).

[b15-cpath-1-2008-093] Lammert M, Mautner VF, Kluwe L (2005). Do hormonal contraceptives stimulate growth of neurofibromas? A survey on 59 NF1 patients. BMC Cancer.

[b16-cpath-1-2008-093] McLaughlin EM, Jacks T (2003). Progesterone receptor expression in neurofibromas. Cancer Res.

[b17-cpath-1-2008-093] Posma E, Aalbers R, Kurniawan YS (2003). Neurofibromatosis type 1 and preganancy: a fatal attraction? Development of malignant Schwannoma during pregnancy in a patient with neurofibromatosis type 1. BJOG: Intern J Obstet Gyn.

[b18-cpath-1-2008-093] Puls LE, Chandler PA (1991). Malignant schwannoma in pregnancy. Acta Obstet Gynecol Scand.

[b19-cpath-1-2008-093] Theos A, Korf BR (2006). Pathophysiology of Neurofibromatosis type 1. Ann. Int. Med.

[b20-cpath-1-2008-093] Weigel NL (1996). Steroid hormone receptors and their regulation by phosphorylation. Biochem J.

[b21-cpath-1-2008-093] Zhu Y, Ghosh P, Charnay P (2002). Neurofibromas in Nf1: Schwann cell origin and role of tumour environment. Science.

